# Dengue Infection as a Trigger for Collapsing Glomerulopathy in Patients With *APOL1* High-Risk Genotype

**DOI:** 10.1016/j.ekir.2025.08.005

**Published:** 2025-08-11

**Authors:** Precil D.M.M. Neves, Luana C.S. Silva, Maria E.V.C. Pereira, Felipe A.M. Oliveira, Stanley A. Araújo, David C. Wanderley, Felipe L. Ledesma, Alekssia L. Guedes, Bárbara H. Loos, Andressa T. Ribeiro, Andréia Watanabe, Elieser H. Watanabe, Gilson M. Murata, Gyl E.B. Silva, José M. Vieira-Júnior, Natália C.V. Melo, Denise M.A.C. Malheiros, Irene L. Noronha, Luiz F. Onuchic

**Affiliations:** 1 Division of Nephrology, University of São Paulo School of Medicine, São Paulo, Brazil; 2 Division of Molecular Medicine, University of São Paulo School of Medicine, São Paulo, Brazil; 3 Nephrology and Dialysis Center, Hospital Alemão Oswaldo Cruz, São Paulo, Brazil; 4 Nephrology Division, Escola Superior de Ciências da Saúde, Brasília, Brazil; 5 Nephrology Division, Hospital Israelista Albert Einstein, São Paulo, Brazil; 6 Electron Microscopy Center, Federal University of Minas Gerais, Belo Horizonte, Minas Gerais, Brazil; 7 Nephropathology Institute, Belo Horizonte, Minas Gerais, Brazil; 8 Pathology Division, University of São Paulo School of Medicine, São Paulo, Brazil; 9 Pediatric Nephrology Division, University of São Paulo, São Paulo, Brazil; 10 Pathology Division, Federal University of Maranhão, São Luís, Maranhão, Brazil; 11 Pathology Division, Hospital Israelista Albert Einstein, São Paulo, Brazil

## Introduction

Collapsing glomerulopathy (CG) is a severe kidney disease associated with fast progression to chronic kidney disease requiring kidney replacement therapy.^1^^,^^2^ Its pathogenesis is multifactorial and may be associated with infections, drugs, autoimmune diseases, and genetic factors, which include genetic susceptibility (*APOL1* high-risk genotypes [*APOL1*-HRG]), or monogenic disorders.^3^

Viruses account for most CG-associated infections, particularly HIV and SARS-CoV-2. In many cases, viral infection may be the trigger to CG development, especially in patients harboring *APOL1*-HRG.^4^^,^^5^

Dengue is a frequent viral disease in tropical and subtropical regions of the world. Its clinical presentation ranges from asymptomatic cases to death.^6^^,^^7^ Although the most common kidney injury affects the tubulointerstitial compartment,^8^ rare cases of glomerulopathies associated with the dengue virus (DENV) have been reported. Herein we report the first 3 cases of CG triggered by DENV in patients with *APOL1*-HRG.

## Case Presentations

### Patient 1

A 54-year-old Black female with a prior 4-day diagnosis of dengue (positive NS1 antigen test) was admitted for fatigue, drowsiness, and facial paralysis initiated 12 hours earlier. She was previously diagnosed with hypertension, diabetes mellitus, and chronic kidney disease stage 4A2 (baseline estimated glomerular filtration rate [eGFR]: 22 ml/min per 1.73 m^2^ and albuminuria: 230 mg/d). Her physical examination revealed a prostrated patient with hypotension (blood pressure: 90/40 mm Hg), left hemiplegia, and right facial paralysis. Encephalic magnetic resonance imaging identified an acute ischemic vascular in the right frontoparietal region.

Laboratory tests showed the following: blood urea nitrogen of 117 mg/dl, creatinine of 11 mg/dl (eGFR: 4 ml/min per 1.73 m^2^), no acid-base or electrolytic abnormalities, hemoglobin of 13.7 g/dl, platelets of 145,000/mm^3^, urinalysis with hematuria, 24-hour proteinuria of 24.4 g, albumin of 1.9 g/dl, total cholesterol of 151 mg/dl, low-density lipoprotein of 122 mg/dl, high-density lipoprotein of 18 mg/dl, triglycerides of 680 mg/dl, complement within the normal range; serologies for HIV, hepatitis B, hepatitis C, cytomegalovirus, Epstein-Barr virus, and autoimmunity workup were negative.

The patient was started on hemodialysis and was treated with methylprednisolone 1 g/d for 3 days and maintained thereafter on prednisone 80 mg/d.

A kidney biopsy was performed 25 days after admission. Light microscopy showed 14 of 18 glomeruli globally sclerotic and 2 glomeruli with segmental synechia. The other 2 viable glomeruli exhibited collapse of capillary loops associated with podocyte hyperplasia and hypertrophy. There was acute tubular necrosis and interstitial fibrosis / tubular atrophy of 60% of the sample. Immunofluorescence revealed C3 deposition (+2/+3) in sclerotic regions. The microscopic findings were consistent with CG. Electron microscopy was not performed because of an absence of viable glomeruli in the remaining tissue.

The patient was steroid-resistant and did not recover kidney function, having progressed to chronic kidney disease requiring kidney replacement therapy. Later-performed genotyping revealed an *APOL1*-HRG (G1/G1).

### Patient 2

A 61-year-old male previously diagnosed with hypertension was referred on the seventh day of dengue diagnosis (positive NS1 antigen test) because of altered laboratory tests. At admission, he was prostrated and exhibited a diffuse rash. Physical examination showed mild dehydration, blood pressure of 109/75 mmHg, and no edema.

Laboratory tests showed the following: blood urea nitrogen of 75 mg/dl, creatinine of 4.6 mg/dl, eGFR of 14 ml/min per 1.73 m^2^ (previous eGFR: 98 ml/min per 1.73 m^2^), no acid-base or electrolytic disorders, hemoglobin of 13.9 g/dl, and platelets of 99,000/mm^3^. Urinalysis revealed hematuria and 24-hour proteinuria of 2.64 g. Total cholesterol was 140 mg/dl, low-density lipoprotein was 75 mg/dl, high-density lipoprotein was 40 mg/dl, albumin was 3.0 g/dl, C3 and C4 were within the normal ranges; serologies for HIV, hepatitis B, hepatitis C, cytomegalovirus and Epstein-Barr virus, as well as autoimmunity workup were negative.

The patient was submitted to a kidney biopsy. Light microscopy identified 3 of 28 glomeruli exhibiting segmental synechia and 5 displaying collapse of capillary loops associated with podocyte hypertrophy and hyperplasia, in addition to acute tubular necrosis. Immunofluorescence revealed IgM and C3 (1+/3+) in sclerotic areas. Electron microscopy identified effacement of foot processes in 60% of the capillary loops, and an absence of tubuloreticular inclusions. The findings were consistent with CG.

No immunosuppression was prescribed. His kidney function improved progressively, and his urine protein levels decreased spontaneously. Genotyping for *APOL1* identified an HRG (G1/G1). His last creatinine was 1.2 mg/dl and 24-hour proteinuria of 0.4g, with use of angiotensin II receptor blocker.

### Patient 3

A 41-year-old female, previously diagnosed with sickle cell anemia, hypertension, and chronic kidney disease stage 3A/A3 (baseline eGFR: 48 ml/min per 1.73 m^2^ and albuminuria: 500 mg/d) was admitted with a 6-day history of fever, headache, and reduced urine output. Her NS1 dengue test was positive. Upon physical examination, she had edema and a blood pressure of 145/79 mm Hg.

Laboratory tests showed the following: blood urea nitrogen of 39 mg/dl, creatinine of 6.7 mg/dl (eGFR: 7.4 ml/min per 1.73 m^2^), no acid-base or electrolytic disorders, hemoglobin of 7.8 g/dl, platelets of 65,000/mm^3^, albumin of 2.5 g/dl. Total cholesterol was 147 mg/dl, low-density lipoprotein was 83 mg/dl, and high-density lipoprotein was 17 mg/dl. Urinalysis revealed hematuria and 24-hour proteinuria of 50g. C3 and C4 were within the normal ranges; serologies for HIV, hepatitis B, hepatitis C, cytomegalovirus, and Epstein-Barr virus, as well as autoimmunity workup were negative.

A kidney biopsy was performed. Light microscopy revealed 6 of 17 glomeruli globally sclerotic, with global collapse of the capillary loop, and podocyte hyperplasia and hypertrophy, interstitial fibrosis / tubular atrophy of 30% of the compartment. Immunofluorescence showed C3 (2+/3+) in sclerotic areas. Electron microscopy revealed effacement of foot processes in 80% of the capillary loops, and an absence of tubuloreticular inclusions. These findings were consistent with CG. The patient harbored an *APOL1*-HRG (G1/G1).

Hemodialysis was initiated and the patient was treated with methylprednisolone 500 mg for 3 days, followed by prednisone 80 mg/d and mycophenolate mofetil 2 g/d. The patient was still on kidney replacement therapy 1 month after starting treatment.

## Discussion

DENV is an RNA flavivirus transmitted by the *Aedes aegypti* and *A albopictus* mosquitoes.^6^^,^^7^ It is one of the most prevalent arboviruses worldwide, with an epidemic in Latin America in 2024.^9^

**Figure 1:**
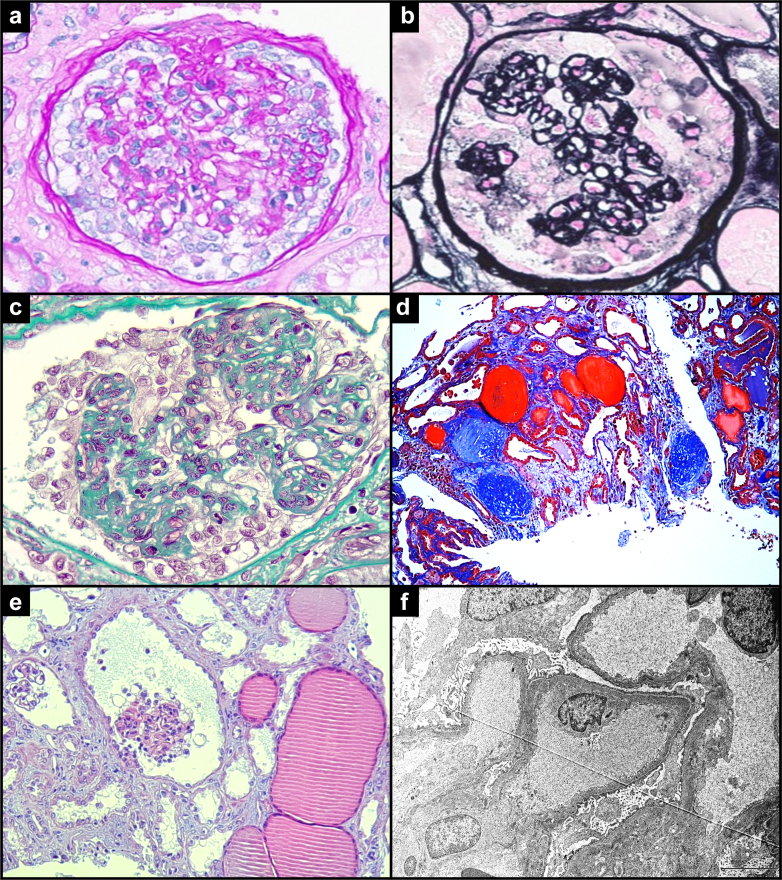
Kidney biopsy from collapsing glomerulopathy triggered by Dengue virus infection. **(a**) Segmental glomerular collapse associated with podocyte hypertrophy or hyperplasia (periodic acid–Schiff, 400×). (b) Glomerular tuft collapse and podocyte hypertrophy/hyperplasia (methenamine silver, 400×). (c) Glomerulus with podocyte hypertrophy or hyperplasia and tuft retraction (Masson’s Trichrome, 400×). (d) Trichrome stain showing segmental sclerosis with interstitial fibrosis and mild tubular atrophy (Masson’s Trichrome, 100×). (e) Collapsed capillary tuft with podocyte hypertrophy and hyperplasia beside microcystic tubular dilatation (hematoxylin and eosin, 200×). (f) Electron microscopy revealing diffuse podocyte foot process effacement.

Dengue is associated with a broad clinical presentation ranging from asymptomatic form, dengue fever, dengue hemorrhagic fever, or dengue shock syndrome, which is a life-threatening condition.^6^^,^S1 Kidney involvement is observed in approximately 15% of the patients, most often as acute kidney injury. This injury is characterized by tubulointerstitial damage manifested as acute tubular necrosis, which may be caused by a direct viral effect, hypovolemia, and/or rhabdomyolysis.S2^,^S3 Glomerular injuries, such as CG (Figure 1), immune-complex-mediated membranoproliferative glomerulonephritis, diffuse proliferative glomerulonephritis, and vascular injuries, such as diffuse cortical necrosis and thrombotic microangiopathy are rare.S4–S8

The mechanism of *APOL1*-mediated kidney diseases follows a “2-hit” process. The first hit consists of harboring an *APOL1*-HRG, and the second hit is the exposure to a trigger that initiates the disease process. This trigger may be infectious, autoimmune or a drug.S9 Under stimuli of interferon and toll-like receptor agonists, *APOL1* transcription is upregulated at the cellular level, leading to a number of cell damaging mechanisms.S10 Interestingly, the recognition of DENV by the immunologic system leads to stimulation of toll-like receptor 3 and toll-like receptor 7. These receptors activate intracellular inflammatory pathways, such as the NF-κB pathway, ultimately activating transcription of inflammatory cytokines genes such as *INF-α*, *TNF-α*, and *IL-6*. Of note, interferon type I produced in this setting inhibits infection of other monocytes by DENV through progressive activation of the JAK/STAT pathway.S11^,^S12 In this scenario, viral recognition and immunologic response to DENV activate common pathways to *APOL1*-mediated kidney diseases. Dengue pathogenesis therefore strongly supports dengue infection as a potential second hit for *APOL1*-mediated kidney diseases, triggering the disease in the setting of *APOL1*-HRG (Table 1).

**Table 1:** Teaching points

• CG is a severe kidney disease which may be associated with infections, drugs, autoimmune diseases, mitochondrial dysfunction, and genetic factors.
• Viral infections, such as HIV and SARS-CoV-2 are frequently associated with CG.
• The inheritance patterns of genetic diseases associated with CG include genetic susceptibility (*APOL1* high-risk genotypes) and monogenic disorders.
• Our cases of DENV-associated CG highlight the relevance of *APOL1* genotyping in cases of CG associated with viral infections, because there is a strong connection between these 2 conditions.

CG, collapsing glomerulopathy; DENV, dengue virus; SARS-CoV-2, severe acute respiratory syndrome-coronavirus 2.

Araujo *et al.*S5 described 11 Brazilian patients diagnosed with CG following dengue infection. This was the first report of such an association. Six of them were genotyped for *APOL1* but all resulted negative. Another Brazilian report describes 2 cases of CG associated with DENV.S4 These patients were initially treated with corticosteroids for a short time and maintained on an angiotensin-converting enzyme inhibitor, having displayed partial remission of proteinuria and partial recovery of kidney function. However, *APOL1* genotyping was not performed in these cases. Patient 2 underwent an atypical progression of CG associated with *APOL1*-HRG. After an initial worsening of kidney function, he maintained stable creatinine values. In turn, the creatinine and proteinuria values started to decrease 1 week after the diagnosis. In this scenario, we opted not to treat the patient with immunosuppressive drugs. We suggest that the following 2 conditions may explain this phenomenon: (i) dengue is an acute and self-limiting disease, so there is no frequent stimulus from the virus to perpetuate the disease; and (ii) the patient had no other previous kidney disease or kidney dysfunction when infected by DENV.

Little data regarding *APOL1*-HRG in Brazil have been reported to date, so there is not an estimation on the frequency of the risk alleles in the general population. Studies on this topic including patients undergoing dialysisS13^,^S14 and pediatric patients with nephrotic syndromeS15^,^S16 revealed a prevalence of up to 9%. When we focus on CG, a previous study from our groupS9 revealed that almost half of the patients diagnosed with idiopathic CG harbored *APOL1*-HRG. In addition, unpublished data from our group suggest that the CG-*APOL1*-HRG association may be even higher in CG cases associated with infections, such as HIV and COVID-19. Because Brazil is a highly admixed country, self-declared race has poor correlation with *APOL1*-HRG.

In conclusion, we report the first 3 cases of CG associated with dengue infection in patients with *APOL1*-HRG. This emphasizes the relevance of *APOL1* genotyping in cases of podocytopathies associated with viral infections.

## Disclosure

All the authors declared no competing interests.

## Patients Consent

The authors declare that they have obtained consent from the patients discussed in the report.

### Funding

This work was supported by the 10.13039/501100001807Fundação de Amparo à Pesquisa do Estado de São Paulo (2020/02988-7 to LFO).

## Supplementary Material

Supplementary File (PDF) **Supplementary References**.
