# Supplementary material for: Dengue Infection as a Trigger for Collapsing Glomerulopathy in Patients With APOL1 High-Risk Genotype
Source: Kidney Int Rep. 2025 Aug 11;10(11):4101–5. doi: 10.1016/j.ekir.2025.08.005 (PMC12640018; doi:10.1016/j.ekir.2025.08.005)
Supplement: Supplementary File (PDF) — Supplementary References. [file mmc1.pdf]

SUPPLEMENTARY REFERENCES

S1. Kularatne SA, Dalugama C. Dengue infection: Global importance, immunopathology and management. *Clinical Medicine*. 2022;22(1):9-13. doi:10.7861/clinmed.2021-0791

S2. Bignardi PR, Pinto GR, Boscarioli MLN, Lima RAA, Delfino VDA. Acute kidney injury associated with dengue virus infection: a review. Brazilian Journal of Nephrology. 2022;44(2):232-237. doi:10.1590/2175-8239-jbn-2021-0221

S3.Gurugama P, Jayarajah U, Wanigasuriya K, Wijewickrama A, Perera J, Seneviratne SL. Renal manifestations of dengue virus infections. Journal of Clinical Virology. 2018;101:1-6. doi:10.1016/j.jcv.2018.01.001

S4. Queiroz PC, Jorge AES, Mourão PHV, Penido MGMG. Collapsing focal segmental glomerulosclerosis probably triggered by dengue virus infection - two case reports. Brazilian Journal of Nephrology. 2020;42(4):489-493. doi:10.1590/2175-8239-jbn-2019-0237

S5. Araújo S de A, Cordeiro TM e, Belisário AR, et al. First report of collapsing variant of focal segmental glomerulosclerosis triggered by arbovirus: dengue and Zika virus infection. Clin Kidney J. 2019;12(3):355-361. doi:10.1093/ckj/sfy104

S6.Alobaidi S, Bali H, Tungekar MF, Akl A. Dengue Virus Infection Presenting as Membranoproliferative Glomerulonephritis Type 1. Cureus. 2021;13(4):e14294. doi:10.7759/cureus.14294

S7. Coelho Júnior JL, Israel KCP, Machado CEE, et al. Thrombotic microangiopathy associated with arboviral infection: Report of 3 cases. Messer WB, ed. PLoS Negl Trop Dis. 2021;15(10):e0009790. doi:10.1371/journal.pntd.0009790

S8. Rodríguez-Iturbe B, Burdmann EA, Barsoum RS. Glomerular Diseases Associated with Infection. Comprehensive Clinical Nephrology. 2010:662–74. doi: 10.1016/B978-0-323-05876-6.00055-1

S9. Neves PD, Watanabe A, Watanabe EH, et al. Idiopathic collapsing glomerulopathy is associated with APOL1 high-risk genotypes or Mendelian variants in most affected individuals in a highly admixed population. Kidney Int. 2024;105(3):593-607. doi:10.1016/j.kint.2023.11.028

S10. Nichols B, Jog P, Lee JH, et al. Innate immunity pathways regulate the nephropathy gene Apolipoprotein L1. Kidney Int. 2015;87(2):332-342. doi:10.1038/ki.2014.270

S11. Uno N, Ross TM. Dengue virus and the host innate immune response. Emerg Microbes Infect. 2018;7(1):1-11. doi:10.1038/s41426-018-0168-0

S12. Nanaware N, Banerjee A, Mullick Bagchi S, Bagchi P, Mukherjee A. Dengue Virus Infection: A Tale of Viral Exploitations and Host Responses. Viruses. 2021;13(10):1967. doi:10.3390/v13101967

S13. Riella C, Siemens TA, Wang M, Campos RP, Moraes TP, Riella LV et al. APOL1-Associated Kidney Disease in Brazil. Kidney Int Rep. 2019;4(7):923-929. doi: 10.1016/j.ekir.2019.03.006.

S14. Alladagbin DJ, Fernandes PN, Tavares MB, Brito JT, Oliveira GGS, Silva LK, et al. The sickle cell trait and end stage renal disease in Salvador, Brazil. PLoS One. 2018;13(12):e0209036. doi: 10.1371/journal.pone.0209036.

S15. Watanabe A, Miranda de Menezes Neves PD, Nunes K, Lerario AM, Watanabe EH, Ferreira FM, et al. Steroid-Resistant Nephrotic Syndrome Is Associated With a Unique Genetic Profile in a Highly Admixed Pediatric Population. Kidney Int Rep. 2024;9(12):3501-3516. doi: 10.1016/j.ekir.2024.09.005.

S16. Watanabe A, Guaragna MS, Belangero VMS, Casimiro FMS, Pesquero JB, de Santis Feltran L, et al. *APOL1* in an ethnically diverse pediatric population with nephrotic syndrome: implications in focal segmental glomerulosclerosis and other diagnoses. Pediatr Nephrol. 2021;36(8):2327-2336. doi: 10.1007/s00467-021-04960-w.
